# Comparing Methods for Calculating Nano Crystal Size of Natural Hydroxyapatite Using X-Ray Diffraction

**DOI:** 10.3390/nano10091627

**Published:** 2020-08-19

**Authors:** Marzieh Rabiei, Arvydas Palevicius, Ahmad Monshi, Sohrab Nasiri, Andrius Vilkauskas, Giedrius Janusas

**Affiliations:** 1 Faculty of Mechanical Engineering and Design, Kaunas University of Technology, LT-51424 Kaunas, Lithuania; marzieh.rabiei@ktu.edu (M.R.); andrius.vilkauskas@ktu.lt (A.V.); 2 Department of Materials Engineering, Isfahan University of Technology, Isfahan 84154, Iran; 3 Department of Polymer Chemistry and Technology, Kaunas University of Technology, LT-50254 Kaunas, Lithuania

**Keywords:** nanocrystal size, x-ray diffraction, Scherrer equation, hydroxyapatite, BET, TEM

## Abstract

We report on a comparison of methods based on XRD patterns for calculating crystal size. In this case, XRD peaks were extracted from hydroxyapatite obtained from cow, pig, and chicken bones. Hydroxyapatite was synthesized through the thermal treatment of natural bones at 950 °C. XRD patterns were selected by adjustment of X-Pert software for each method and for calculating the size of the crystals. Methods consisted of Scherrer (three models), Monshi–Scherrer, three models of Williamson–Hall (namely the Uniform Deformation Model (UDM), the Uniform Stress Deformation Model (USDM), and the Uniform Deformation Energy Density Model (UDEDM)), Halder–Wanger (H-W), and the Size Strain Plot Method (SSP). These methods have been used and compared together. The sizes of crystallites obtained by the XRD patterns in each method for hydroxyapatite from cow, pig, and chicken were 1371, 457, and 196 nm in the Scherrer method when considering all of the available peaks together (straight line model). A new model (straight line passing the origin) gave 60, 60, and 53 nm, which shows much improvement. The average model gave 56, 58, and 52 nm, for each of the three approaches, respectively, for cow, pig, and chicken. The Monshi–Scherrer method gave 60, 60, and 57 nm. Values of 56, 62, and 65 nm were given by the UDM method. The values calculated by the USDM method were 60, 62, and 62 nm. The values of 62, 62, and 65 nm were given by the UDEDM method for cow, pig, and chicken, respectively. Furthermore, the crystal size value was 4 nm for all samples in the H-W method. Values were also calculated as 43, 62, and 57 nm in the SSP method for cow, pig, and chicken tandemly. According to the comparison of values in each method, the Scherrer method (straight line model) for considering all peaks led to unreasonable values. Nevertheless, other values were in the acceptable range, similar to the reported values in the literature. Experimental analyses, such as specific surface area by gas adsorption (Brunauer–Emmett–Teller (BET)) and Transmission Electron Microscopy (TEM), were utilized. In the final comparison, parameters of accuracy, ease of calculations, having a check point for the researcher, and difference between the obtained values and experimental analysis by BET and TEM were considered. The Monshi–Scherrer method provided ease of calculation and a decrease in errors by applying least squares to the linear plot. There is a check point for this line that the slope must not be far from one. Then, the intercept gives the most accurate crystal size. In this study, the setup of values for BET (56, 52, and 49 nm) was also similar to the Monshi–Scherrer method and the use of it in research studies of nanotechnology is advised.

## 1. Introduction

A crystallite solid is defined as an aggregate involving of atoms, molecules, or ions accumulated together in a periodic arrangement [1]. Disorder of the discipline and periodicity of the constituent can be created in crystalline solids, which the terms “order” and “disorder” are cited to the collective nature or degree of such disturbance [2]. XRD profile analysis is a convenient and powerful method to investigate crystallite size and lattice strain. Utilizing X-ray patterns and crystallography is an easy way for calculating the size of nanocrystallites, especially in nanocrystalline bulk materials. Paul Scherrer published his paper [3] and introduced the Scherrer equation in 1918. In addition, Uwe Holzwarth and Neil Gibson announced that the Scherrer equation is related to a sharp peak of X-ray diffraction. The equation was introduced with the subscript (hkl) because it is related to the one peak only. It is important to note that the Scherrer equation can only be utilized for average sizes up to around 100 nm. It also depends on the instrument, as well as relationship between signal and sample to criterion noise, because when crystallite size increases, diffraction peak broadening decreases. It can be very hard to provide separation and distinguishing of broadening through the crystallite size from the broadening due to other parameters and factors. Errors always exist and successful calculation methods are those which can decrease the errors in the best possible way to yield more accurate data. Calculation of nanoparticle size extracted by XRD patterns is not possible because a particle has several nanoscale or microscale crystals. An X-ray can penetrate through the crystal size to provide information, therefore, the calculation of size is not related to the particles and is related to the crystals. In this study, three samples of hydroxyapatite obtained from natural bones of cow, pig, and chicken are used to obtain peak lists of XRD patterns. The aim and novelty of this study is comparison of all methods available for X-ray diffraction (XRD peaks) for finding the size of crystals, especially for natural hydroxyapatite. Seven methods related to the XRD peaks are used, calculated, and discussed. A new model for calculation in the Scherrer method (straight line passing the origin) is presented. Nowadays, there is increasing interest in different fields of nanomaterials such as tissue engineering. One of these is the hydroxyapatite from biological sources such as bovine due to their different applications [4]. In this case, hydroxyapatite obtained from cow, pig, and chicken bones was selected. Size of hydroxyapatite crystals in animal bones is interesting for fundamental and applied sciences such as doping metals, bioglass, polymers, and composites to hydroxyapatite specially for fabrication implants.

## 2. Materials and Experiments

Natural bones of cow, pig and chicken were prepared from a Maxima LT shop (according to the EU Regulation—Lithuanian breeds) and they were first boiled in hot water for two hours to eliminate meats and fats on the surface of the bones. Then, the bones were cleaned and dried at 110 °C for two hours. Finally, thermal treatment of hydroxyapatite was performed in a furnace at 950 °C for two hours to allow diffusion of proteins, such as collagens, from inside of bones to the surface, and burning at high temperatures. The model of the furnace was E5CK-AA1-302 (Snol 6, 7/1300). In this study, D8 Discover X-ray diffractometer (Bruker AXS GmbH, Kaunas, Lithuania) with Cu_Kα_ radiation was used. A white and clean hydroxyapatite was obtained. Then, samples were grinded in a rotary ball mill with some volume ratios of fired grogs, steel balls, and empty space. The model of the ball mill was planetary Fritsch Pulverisette-5 (Kaunas, Lithuania). The particle sizes were micron scale, while the crystal sizes inside the particles were nanosize, as it is known in the literature [5]. The powder X-ray diffractions were taken at 40 kV and 40 mA, and recorded from 20 to 50 degrees for 2θ at a scanning speed of 2.5 degrees/minute and a step size of 0.02 degrees. The resulting patterns were studied by version 4.9 of High Score X’Pert software analysis, which uses the fundamental parameter procedure implemented in ASC suffix files. In addition, the specific surface area of the samples was measured by desorption isotherms of nitrogen (N_2_) gas through the use of a Brunauer–Emmett–Teller (BET) apparatus Gemini V analyzer, micrometrics GmbH, (Isfahan, Iran) For chemical elements of the samples, an energy dispersive X-ray (EDX) spectrometer Phillips/FEI Quanta 200 was utilized. In addition, for thin layers of the samples, the transmission electron microscopy (TEM), CM 10-Philips (Tehran, Iran) with acceleration voltage between 50 and 80 KV, was used.

### 2.1. Preparation of Hydroxyapatite Powders

**Figure 1:**
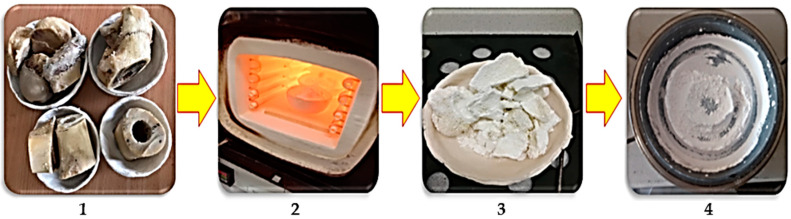
Images of production route of hydroxyapatite obtained from cow, pig, and chicken bones (steps **1**–**4**).

**Figure 2:**
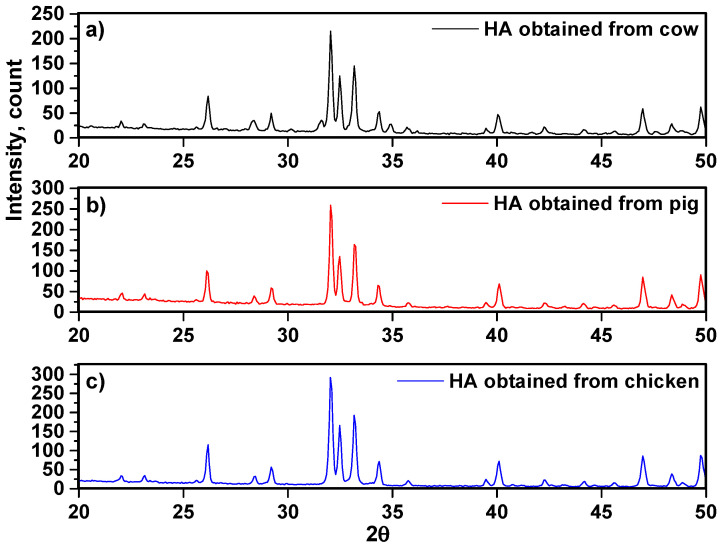
XRD patterns of hydroxyapatite obtained from (**a**) cow, (**b**) pig, and (**c**) chicken bones.

Femur bones of cow, pig, and chicken were prepared. Bovine bones were first separated and boiled in hot water, and then, immersed into acetone for two hours to remove collagen and fat (step 1, Figure 1). In step 2, bones were washed by distillated water and dried two times. Then, bones were placed in separate steps into the furnace under ambient conditions and the rate of increasing temperature was 10 °C/minute. Finally, bones were fired at 950 °C for 2 h and they were cooled inside the furnace very slowly. Following this process, the first black fired bones (due to carbon release) turned into a white granular bulk. Furthermore, bones were transformed to fully crystallized hydroxyapatite at 950 °C (step 3) [6]. Hydroxyapatite extracted from cow, pig, and chicken bones was placed into a planetary ball mill device involving a bowl (tungsten carbide) and balls, to fabricate fine particles after heat-treating. The feed ratio was 30 g powder to 300 g of balls (1 to 10 weight ratio), the speed was fixed at 250 rpm, and the milling time adjusted at 2 h with pause and reverse mode (step 4), according to the procedure described in literatures [7,8]. The images of production route of hydroxyapatite are presented in Figure 1. The phase composition and purity of the materials were determined by X-ray diffraction (XRD). The XRD patterns of the hydroxyapatite white powder produced after milling are presented in Figure 2. The XRD patterns were investigated completely through the X’Pert software and patterns were confirmed via standard XRD peaks of hydroxyapatite based on ICDD 9-432. Similar observations have been reported by Bahrololoom and Shahabi [9,10]. In addition, crystallographic parameters of each individual XRD pattern are presented in Table 1, Table 2 and Table 3, respectively. Moreover, crystallographic parameters related to the structures resulting from X’Pert software analysis could be seen in Table 4. The unit cell parameters were in good agreement with the results corresponded by other researchers for fabrication of hydroxyapatite [11,12].

### 2.2. XRD Analysis of Samples

According to the XRD patterns (Figure 2), it is observed that the crystallization of hydroxyapatite samples was nearly similar. The pattern of XRD is shown at angles between 20° < 2θ < 50°. The largest peaks are observed, corresponding to crystalline hydroxyapatite at around 31.96°, 32.04°, and 32.03° for cow, pig, and chicken, respectively. Based on the pattern, the strong diffraction peaks at 2θ values are attributed to the hydroxyapatite structure, whose hkl values of exact hydroxyapatite peaks are related to the 002, 102, 210, 211, 112, 300, and 202, respectively [13]. In addition, the values of full width at half maximum of the peaks (β) in radians were recorded in the range of 0.00174 to 0.00348, 0.00226 to 0.00313, and 0.00244 to 0.00313 for hydroxyapatite obtained from cow, pig, and chicken bones, respectively (Table 1, Table 2 and Table 3). Furthermore, the maximum intensity of samples was not different and the count was in the range of ~250 counts. The reason is related to the same generation and nature of hydroxyapatite samples.

## 3. Results and Discussions

### 3.1. Scherrer Method

The Scherrer equation relates to the diffraction peak submitted in Equation (1) [3], where L is the nanocrystal size; K is the shape factor, usually taken as 0.89 for ceramic materials; λ is the wavelength of radiation in nanometer ($\lambda_{{\mathrm{Cu}}_{K\alpha }}$ = 0.15405 nm); θ is the diffracted angle of the peak; β is the full width at half maximum of the peak in radians. In addition, broadening in the peaks is related to physical broadening and instrumental broadening [14,15].

(1) $$L\;=\;\frac{K\lambda }{\beta }.\frac{1}{\cos\theta }$$

For decreasing this error of instrument, Equation (2) can be used:

(2) $$\beta_{d}^{2}\;=\;\beta_{m}^{2}\;-\;\beta_{i}^{2}$$

In this formula, β_m_ is the measured broadening, β_i_ is the instrumental broadening, and β_d_ was introduced as the corrected broadening responsible for crystal size. Furthermore, in this case, crystalline silicon was used as the reference material for calibration of instrumental error. The instrumental broadening and physical broadening of the sample measured through the full width half maximum (FWHM) and with utilizing the correction of physical broadening, it will be possible to follow up calculation on the crystal size with the Scherrer equation, such as cited in [16,17]. There are several publications that used calculation of the Scherrer equation only for the sharpest peak and they were not considering calculations for all or selected peaks.

#### 3.1.1. Straight Line Model in Scherrer Method

**Figure 3:**
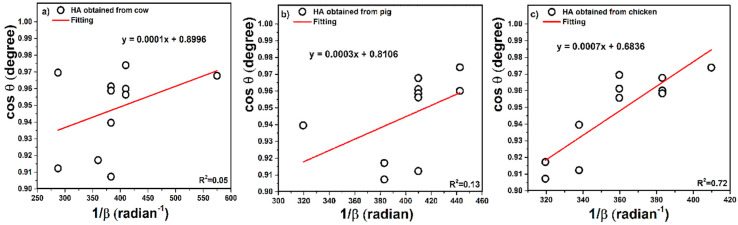
Pattern of XRD analysis of hydroxyapatite obtained from (**a**) cow, (**b**) pig, and (**c**) chicken bones.

In this case, all peaks were considered and according to the Scherrer equation, plots of cosθ versus 1/β (inverse radian unit) for the samples are presented in Figure 3, respectively. This is a straight line model to provide the possibility of using all or selected peaks simultaneously.

According to Equation (3), the slope of plots is equal to $\frac{K\lambda }{L}$, therefore, the values of slope reported as 0.0001 for cow, 0.0003 for pig, and 0.0007 for chicken tandemly, after the calculation, gave values of crystal size as 1371, 457, and 196 nm for cow, pig, and chicken, respectively.

(3) $$\cos\theta \;=\;\frac{K\lambda }{L}.\frac{1}{\beta }$$

It is obvious that when hydroxyapatite from natural bone is naturally nanocrystal, the values of crystallite size calculated from the slope of the linear fit are invalid, since they all should be under 100 nm. It is assumed that when the least squares method is applied to fit the data according to the Scherrer equation (Equation (3)), then, the y-intercept in this fit has no physical meaning. In order to correct the use of the Scherrer equation, it is recommended to force the linear plot to pass through the origin.

#### 3.1.2. Model of Straight Line Passing the Origin in Scherrer Method

This is a new model developed in this study. In order to force the linear plot to pass through the origin and obtain a reasonable slope for calculations, Equation (4) [18] was considered. In this equation, all points (Figure 3) were extracted as the plot of y versus x points and the points are presented in Table 5.

(4) $$\mathrm{Slope}\;=\;\frac{x_{1}y_{1}+x_{2}y_{2}+x_{3}y_{3}+\cdots \ldots \ldots +x_{n}y_{n}}{x_{1}^{2}+x_{2}^{2}+x_{3}^{2}+\ldots \ldots \ldots \ldots +x_{n}^{2}}$$

After the calculations, the slope values obtained were 0.0023, 0.0023, and 0.0026 for the samples, therefore, the crystal size was calculated as 60, 60, and 53 nm for cow, pig, and chicken, respectively. This is a modification obtained in this study for the use of the Scherrer equation for all of the peaks simultaneously.

#### 3.1.3. Average Model in Scherrer Equation

An average model on the Scherrer equation was utilized; the crystal size was calculated from Equation (1) and then, averaged. Values of crystal size extracted by the average method based on the Scherrer equation are presented in Table 6. The average values for cow, pig, and chicken are, respectively, 56, 58, and 52 nm. Consequently, it seems that the crystallite size estimated from the slope by the modification of linear fit obtained in this study (case 2) is more consistent than using the Scherrer equation for all of the peaks individually and obtaining the average. This might be due to the fact that as the angle of diffraction increases from 25 to 50 (Figure 2), the values of FWHM become less accurate [19], while taking the average assumes the same validity for all of the points. Applying Equation (4) [18] is a least squares approach for a linear plot that must go through the origin, so that adjustment is applied to decrease the sources of errors.

### 3.2. Modified Scherrer Equation (Monshi–Scherrer Method)

Monshi et al. in 2012 employed some modifications in use of the Scherrer equation and introduced the following formula (Equation (5)) [19]:

The Scherrer equation systematically shows increased values of nanocrystalline size as d (distance of diffracted planes) values decrease and 2θ values increase, since β.cosθ cannot be maintained as constant. Furthermore, the Modified Scherrer equation can provide the advantage of decreasing the errors or $\Sigma \;{\left(\pm \Delta \ln\beta \right)}^{2}$ to give a more accurate value of L from all or some of the different peaks [19].

(5) $$\mathrm{Ln}\;\beta \;=\;\mathrm{Ln}\;(\frac{K\lambda }{L})\;+\;\mathrm{Ln}\;(\frac{1}{\mathrm{Cos}\;\theta })$$

**Figure 4:**
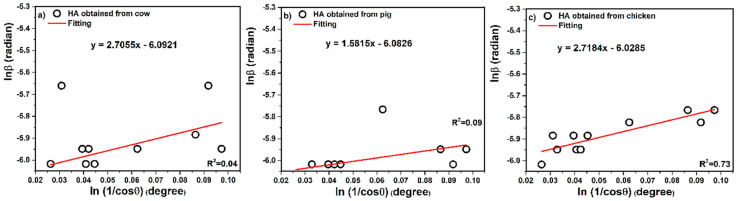
Linear plots of the modified Scherrer (Monshi–Scherrer) equation and gained intercepts for different hydroxyapatites obtained from (**a**) cow, (**b**) pig, and (**c**) chicken bones.

So that the linear plot of Ln β (β in radians) versus Ln $\left(\frac{1}{\mathrm{Cos}\;\theta }\right)$ (degree) can be a linear plot for all or some of the chosen peaks, the least squares statistical method is used to decrease the sources of errors. After stablishing the most accurate linear plot, the value of Ln $\left(\frac{K\lambda }{L}\right)$ can be obtained from the intercept. The e^(intercept)^ gives $\frac{K\lambda }{L}$, from which a single value of L is obtained from all of the available peaks. Lnβ versus ln(1/cosθ) is demonstrated in the plots of Figure 4, together with the equations of the linear least squares method obtained from the linear regression of data in plots. According to the Monshi–Scherrer equation, for finding the size of the crystals, Equation (6) is employed. When using X’Pert software, it is better for making and using an ASC file of peaks data (with suffix ASC) and obtain the peak list including FWHM, which is related to the fit profile icon (right click on the peak and select fit profile in X’Pert software) to create full fitting in finding β (FWHM). After plotting Equation (5) and obtaining the linear equation for the least squares method of all or some selected peaks, then,

(6) $$\frac{K\;\lambda }{L}\;=\;e^{\left(\mathrm{intercept}\right)}$$

Linear equations of hydroxyapatite obtained from cow, pig, and chicken recorded y = 2.7055x − 6.0921, y = 1.5815x − 6.0826, and y = 2.7184x − 6.0285, respectively, and intercept values were −6.0921 for cow, −6.0826 for pig, and −6.0285 for chicken tandemly. Nevertheless, the intercepts were calculated as e^(−6.0921)^ = 0.00227, e^(−6.0826)^ = 0.00228, and e^(−6.0285)^ = 0.00240, respectively. Therefore, $\frac{K\;\lambda }{L}$ = 0.00227, $\frac{K\;\lambda }{L}$ = 0.00228 and $\frac{K\;\lambda }{L}$ = 0.00240 for cow, pig, and chicken tandemly. After the calculations, the values of crystal sizes were obtained as 60, 60, and 57 nm for cow, pig, and chicken, respectively.

Monshi–Scherrer is the only method according to the methods employed in this research that provides a check point for the evaluation of the validity of results. The linear plot of Equation (5) must have a slope of one. Therefore, if it deviates from one, some of the points can be eliminated.

It is explained in this method that if the Scherrer Equation (1) is going to give the same value of crystal size (L) for all of the peaks, then, because Kλ is fixed, β cosθ must be fixed. If cosθ varies from 0.95 to 0.05 as the angle in the XRD test increases, it means 19 times decrease, the FWHM (β) cannot go from 2 mm in computer scale to 38 mm to compensate 19 times increase. This is certainly a source of error. In this study, the points for all of the methods were kept the same for the proper comparison between methods. However, when using the Monshi–Scherrer method, elimination of some of the peaks is advisable to get a slope nearer to one. This decreases sources of errors and gives a more accurate crystal size. This check point can only be assessed in this method. In all other methods, the results should be accepted without any judgement on the validity of obtained data.

### 3.3. Williamson–Hall Method of Analysis

The Scherrer equation focuses only on the effect of crystallite size in XRD peak broadening and it cannot be considered for microstructures of the lattice, i.e., about the intrinsic strain, which becomes developed in the nanocrystals through the point defects, grain boundaries, triple junctions, and stacking faults [20]. One of the methods considering the effect of strain-induced XRD peak broadening is the Williamson–Hall (W-H) method; also, this method provides calculation of the crystal size along with the intrinsic strain [21,22]. According to the physical line broadening of X-ray diffraction peak, it is a combination of size and strain. The W-H method does not confirm a 1/*cosθ* dependency as in the Scherrer equation but varies with *tanθ* in strain considerations. This basic difference pursues a dissociation of broadening reflection and combines small crystallite size and microstrain together. The distinguished θ associations of both effects of size and strain broadening in the analysis of W-H are given as Equation (7).

(7) $$\beta_{\mathrm{total}}=\beta_{\mathrm{size}}+\beta_{\mathrm{strain}}$$

In this case, modified W-H was used and models involved uniform deformation (UDM), uniform stress deformation (USDM), and uniform deformation energy density (UDEDM), which will be discussed.

#### 3.3.1. Uniform Deformation Model (UDM)

The UDM method obtained the following equation (Equation (8)) for the strain associated with the nanocrystals:

(8) $$\varepsilon \;=\;\frac{\beta }{4\;\tan\theta }\;=\;\frac{\beta_{2}\;\cos\theta }{4\;\sin\theta }$$

where β_2_ is the broadening of the width of the peaks due to strain, while the broadening due to nanocrystal size β_1_ comes from the Scherrer equation.

(9) $$\beta \;=\;\beta_{1}\;+\;\beta_{2}\;=\;\frac{K\lambda }{L\;\cos\theta }\;+\;4\varepsilon \;\frac{\sin\theta }{\cos\theta }$$

(10) $$\beta_{\mathrm{hkl}}.\cos\theta \;=\;(\frac{K\lambda }{L})\;+\;(4\varepsilon \;\sin\theta )$$

**Figure 5:**
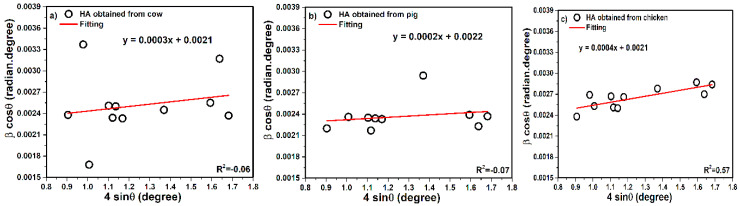
UDM plot of hydroxyapatite obtained from (**a**) cow, (**b**) pig, and (**c**) chicken bones.

According to Equation (10), the term of (β_hkl_ cosθ) corresponds to (4 sinθ) for the preferred orientation peaks of hydroxyapatite with the hexagonal lattice and considers the isotropic nature of the crystals. Figure 5 shows the 4 sinθ as an *X*-axis and β cosθ term along the *Y*-axis. Mostly, UDM is related to an isotropic (perfect) crystal system in all (hkl) planes. Apparently, slope and intercept of the fitted line correspond to the strain and crystal size, respectively. The intercept values equal $\frac{K\lambda }{L}$. The $\frac{K\lambda }{L}$ reported was 0.0021 for cow, 0.0022 for pig, and 0.0021 for chicken. These quantities are estimated from the intercept of the vertical axis and slope, from the plot of β_hkl_ cosθ as a function of 4 sinθ. After calculations, the crystal size values were obtained as 65, 62, and 65 nm for cow, pig, and chicken, respectively. In this plot, the units of 4 sinθ and β cosθ are degree and radian degree tandemly. In addition, several defects influence to the lattice structure via size restriction and it will be caused to the strain lattice. Herein, the slope values (positive values) are represented to the intrinsic strain, therefore, 0.0003 for cow, 0.0002 for pig, and 0.0004 for chicken have been reported. The positive values of intrinsic strain can prove tensile strain and if values were negative, they will be related to the compressive strain.

#### 3.3.2. Uniform Stress Deformation Model (USDM)

For a more realistic crystal system where the anisotropic nature of Young’s modulus is considered [23], there is the generalization of Hooke’s law, where the strain (ε) and stress (σ) are in a linear relationship, with the constant of proportionality being the modulus of elasticity or simply Young modulus. In this method, Hooke’s law was referred to for strain and stress, taking linear proportionality to Equation (11), where σ is stress, ε is strain of the crystal, and E is Young’s modulus respectively.

(11) σ=Eε

This equation is just an access that is credible for a notably small strain. With imaging small strains, Hooke’s law can be utilized. Furthermore, increasing the strain causes deviation of particles from the linear, alternatively [24]. Moreover, for obtaining E, there is Equation (12) and this equation is related to the kind of lattice, for example, in this case, according to the results extracted by X’Pert, hydroxyapatite has hexagonal crystals, therefore, Equation (12) should be used [25]. In this formula, h, k, and l are indexes of the crystallographic plane, and a and c are lattice parameters (these values can be extracted from phase file by X-pert software). In addition, S_11_ S_33_, S_13_, and S_44_ are introduced as elastic compliances and C_11_, C_12_, C_33_, and C_44_ are elastic stiffness constants of hexagonal hydroxyapatite. The values of S_11_, S_33_, S_13_, and S_44_ for hydroxyapatite are presented in Table 7 and the values are cited in reference [26]. In addition, the values of crystallography parameters and Young’s modulus (E) of each individual XRD pattern related to the hydroxyapatite obtained from cow, pig, and chicken are presented in Table 8. In fact, Young’s modulus (E_hkl_) is in the direction perpendicular to the set of crystal lattice planes (hkl).

(12) $$E_{\mathrm{hkl}}=\;\frac{{\left[h^{2}+\frac{{\left(h+2k\right)}^{2}}{3}+{\left(\frac{\mathrm{al}}{c}\right)}^{2}\right]}^{2}}{S_{11}{\left(h^{2}+\frac{{\left(h+2k\right)}^{2}}{3}\right)}^{2}+S_{33}{\left(\frac{\mathrm{al}}{c}\right)}^{4}+\left(2S_{13}+S_{44}\right)\left(h^{2}+\frac{{\left(h+2k\right)}^{2}}{3}\right){\left(\frac{\mathrm{al}}{c}\right)}^{2}}$$

**Figure 6:**
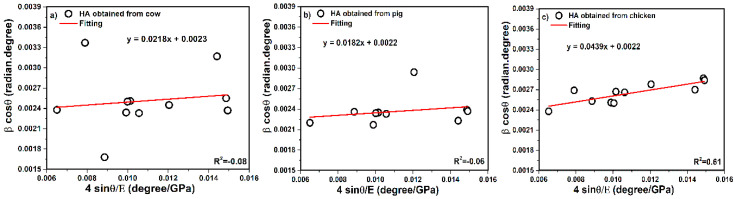
USDM plot of hydroxyapatite obtained from (**a**) cow, (**b**) pig, and (**c**) chicken bones.

According to Equation (13), the terms of $\frac{4\mathrm{Sin}\theta }{E_{\mathrm{hkl}}}$ along the *X*-axis and β_hkl_.cosθ along the *Y*-axis are related to the peaks in the XRD pattern of the samples and are presented in Figure 6.

(13) $$\beta_{\mathrm{hkl}}.\cos\theta \;=\;(\frac{K\lambda }{L})\;+\;4\sigma .\frac{\sin\theta }{E_{\mathrm{hkl}}}$$

The size of crystals has been specified from the USDM method. The intercept values are equal to $\frac{K\lambda }{L}$, therefore, $\frac{K\lambda }{L}$ was reported as 0.0023 for cow, 0.0022 for pig, and 0.0022 for chicken. After calculation, the crystal size values were obtained as 60, 62, and 62 nm for cow, pig, and chicken, respectively. In addition, the slope of the straight line can provide values of stress, nevertheless, the values of stress for cow, pig, and chicken were calculated as 22, 18, and 44 MPa. The state of calculating strain was performed. In this step, average Young’s modulus has further been reported. The average of the E value was calculated as 115.40 for cow, 114.58 for pig, and 115.45 GPa for chicken and values were not far from standard experimental value of Young’s modulus (114 GPa( [26]. Therefore, strain values were calculated as 1.89 × 10^−4^, 1.59 × 10^−4^, and 3.81 × 10^−4^ for cow, pig, and chicken, respectively.

#### 3.3.3. Uniform Deformation Energy Density Model (UDEDM)

The anisotropic energy can be investigated via UDEDM. For knowing the amount of lattice energy saved in the unit volume, we can use a quantity of lattice energy density (LED). In this case, we suppose that the volumetric LED is associated to the effective stiffness of a crystal. As Hooke’s law, LED can be evaluated from Equation (14). Furthermore, stress and strain are related to Equation (11) and the constants in the stress–strain relation are no longer independent when the strain energy density u is taken into account.

(14) $$\mathrm{LED}\;=\;\varepsilon^{2}.\frac{E_{\mathrm{hkl}}}{2}$$

Moreover, the intrinsic strain can be submitted as Equation (15).

(15) $$\varepsilon \;=\;\sigma .\sqrt{\frac{2.\mathrm{LED}}{E_{\mathrm{hkl}}}}$$

Substitution of Equation (15) with Equation (10), yields Equation (16) [27].

(16) $$\beta_{\mathrm{hkl}}.\cos\theta \;=\;(\frac{K\lambda }{L})\;+\;4\sigma .\sin\theta \sqrt{\frac{2.\mathrm{LED}}{E_{\mathrm{hkl}}}}$$

**Figure 7:**
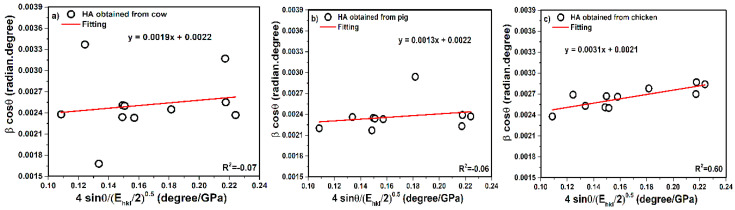
UDEDM plot of hydroxyapatite obtained from (**a**) cow, (**b**) pig, and (**c**) chicken bones.

According to Equation (16), values of crystal size can be calculated, as in Figure 7, $\beta_{\mathrm{hkl}}$.cosθ as a *Y*-axis and the term of $\frac{4\sin\theta }{\sqrt{\frac{E_{\mathrm{hkl}}}{2}}}$ as an *X*-axis.

The intercept values of the plotted straight line equal $\frac{K\lambda }{L}$, therefore, $\frac{K\lambda }{L}$ was reported as 0.0022 for cow, 0.0022 for pig, and 0.0021 for chicken. In the final calculations, the crystal size values were reported as 62, 62, and 65 nm for cow, pig, and chicken tandemly. As a rule, in the application, compounds are not always isotropic, perfect, and homogenous, but also compounds are encountered with defects, agglomeration, dislocations, imperfections, etc. In fact, another model obtained for energy density, where the constants of proportionality corresponded to strain–stress, is considered. In addition, two states similar to the USDM model were considered in calculations.

Moreover, the slope gives the LED, therefore, according to Equations (11) and (14), the values of energy density reported 43.67, 29.70, and 28.28 KJ/m^3^ for cow, pig, and chicken tandemly. For strain values also, states have been noted. According to Equation (14), the strain values were calculated as 0.87 × 10^−3^, 0.73 × 10^−3^, and 0.70 × 10^−3^ (E ~ average Young’s modulus) for cow, pig, and chicken, respectively.

### 3.4. Halder–Wagner Method (H-W)

The fundamental subject of this method involves the assumption that peak broadening is a symmetric Voigt function [28,29]. According to the Voigt function, the full width at half maximum of the physical profile should be considered as Equation (17).

(17) $$\beta_{\mathrm{hkl}}^{2}\;=\;\beta_{L}.\beta_{\mathrm{hkl}}\;+\;\beta_{G}^{2}$$

In this formula, β_L_ and β_G_ are full width at half maximum of the Lorentzian and Gaussian function tandemly. The important observation is the calculation and values of lattice distance between the (hkl) planes (d_hkl_). Hexagonal lattice (hydroxyapatite) is associated with Equation (18), but for cubic crystal lattice distance between the (hkl) planes, (d_hkl_) are corresponded to the Equation (19). The values of d_hkl_ for hydroxyapatite obtained from cow, pig, and chicken bones are presented in Table 1, Table 2 and Table 3.

(18) $$\frac{1}{d_{\mathrm{hkl}}^{2}}\;=\;\frac{4}{3}\left(\frac{h^{2}+\mathrm{hk}+k^{2}}{a^{2}}\right)+\left(\frac{l^{2}}{c^{2}}\right)$$

(19) $$d_{\mathrm{hkl}}^{2}=\left(\frac{a^{2}}{h^{2}+k^{2}+l^{2}}\right)$$

In addition, this method is focused on the peaks at low and middle angles, where the overlapping of diffraction peaks is less. The computation formula of the Halder–Wagner method is presented in Equation (20), as well as subcategories of the formula of this equation cited in Equations (21) and (22) [30].

(20) $${\left(\frac{\beta_{\mathrm{hkl}}^{*}}{d_{\mathrm{hkl}}^{*}}\right)}^{2}\;=\;\frac{1}{L}.\frac{\beta_{\mathrm{hkl}}^{*}}{d_{\mathrm{hkl}}^{*}{}^{2}}\;+\;{\left(\frac{\varepsilon }{2}\right)}^{2}$$

(21) $$\beta_{\mathrm{hkl}}^{*}=\beta_{\mathrm{hkl}}.\frac{\cos\theta }{\lambda }$$

(22) $$d_{\mathrm{hkl}}^{*}=2d_{\mathrm{hkl}}.\frac{\sin\theta }{\lambda }$$

**Figure 8:**
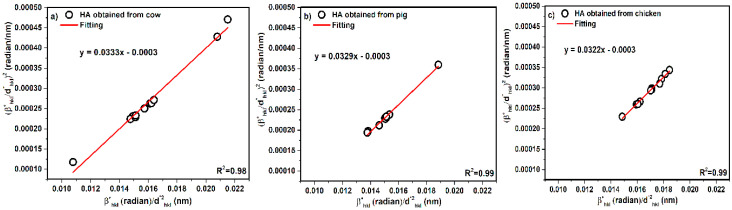
Halder–Wagner plot of hydroxyapatite obtained from (**a**) cow, (**b**) pig, and (**c**) chicken bones.

In addition, d_hkl_ is the lattice distance between the (hkl) planes for the hexagonal crystal, as well as the term of $\frac{\beta_{\mathrm{hkl}}^{*}}{d_{\mathrm{hkl}}^{*}{}^{2}}$ for the *X*-axis and the term of ${\left(\frac{\beta_{\mathrm{hkl}}^{*}}{d_{\mathrm{hkl}}^{*}}\right)}^{2}$ for the *Y*-axis illustrated in Figure 8.

The slope of the plotted line provides calculation of crystal size of samples. The slope values are proportional to $\frac{K\lambda }{L}$ = 0.0333 for cow, $\frac{K\lambda }{L}$ = 0.0329 for pig, and $\frac{K\lambda }{L}$ = 0.0322 for chicken; after calculation, the crystal size values were obtained as 4, 4, and 4 nm for cow, pig, and chicken, respectively. In addition, calculated values of strain from the intercept of the plot equal ${\left(\frac{\varepsilon }{2}\right)}^{2}$, but according to the negative intercept, the following calculation of strain was not possible. 

### 3.5. Size Strain Plot Method (SSP)

In this method, less weight is given to data from reflections at high angles. This has a better result for isotropic broadening, because at higher angles and higher diffracting, XRD data are of lower quality and peaks are overlapped. In this assumption, it is stated that the profile is illustrated by strain profile through the Gaussian function and the crystallite size via Lorentzian function [31]. Furthermore, total broadening of this method was expressed by Equation (23).

β_hkl_ = β_L_ + β_G_ (23)

where, β_L_ and β_G_ are the peak broadening via Lorentz and Gaussian functions tandemly. Equation (24) is the submitted formula of the SSP method [27].

(24) $${\left(d_{\mathrm{hkl}}.\beta_{\mathrm{hkl}}.\cos\theta \right)}^{2}=\;\frac{K\lambda }{L}.\left(d_{\mathrm{hkl}}^{2}.\beta_{\mathrm{hkl}}.\cos\theta \right)+\;\frac{\varepsilon^{2}}{4}$$

**Figure 9:**
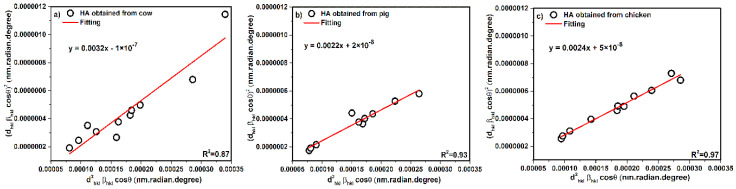
SSP plot of hydroxyapatite obtained from (**a**) cow, (**b**) pig and (**c**) chicken bones.

Figure 9 shows a plot of the $d_{\mathrm{hkl}}^{2}.\beta_{\mathrm{hkl}}.\cos\theta$ term along the *X*-axis and ${\left(d_{\mathrm{hkl}}.\beta_{\mathrm{hkl}}.\cos\theta \right)}^{2}$ along the *Y*-axis corresponding to diffraction peaks.

The slope values are equal to $\frac{K\lambda }{L}$. The $\frac{K\lambda }{L}$ values are reported as 0.0032 for cow, 0.0022 for pig, and 0.0024 for chicken. After calculations, the crystal size values were obtained as 43, 62, and 57 nm for cow, pig, and chicken, respectively. According to Equation (24), the intercept gives the intrinsic strain and the values of intercept are equal to $\frac{\varepsilon^{2}}{4}$, therefore, calculation of intrinsic strain values are possible for pig and chicken samples only because the intercept of cow is negative. The intrinsic strain values were calculated as 2.83 × 10^−4^ and 4.48 × 10^−4^ for pig and chicken, respectively.

### 3.6. Specific Surface Area by Gas Adsorption (BET Method)

The most widely used technique for estimating specific surface area is the BET method. Under normal atmospheric pressure and at the boiling temperature of liquid nitrogen, the amount of nitrogen adsorbed in relationship with pressure gives the specific surface area of powder. The observations are interpreted following the model of the BET method. The samples were degassed at 200 °C under reduced pressure (13 × 10^−7^ atmosphere) for around 15 to 20 h before each measurement. The amount of nitrogen was by volume adsorption at −197 °C. The reported surface area for a bone-derived hydroxyapatite is much lower and the value is around 0.1 m^2^/g [32]. However, one synthetic hydroxyapatite (not sintered or deproteinized bone) is 17 to 82 m^2^/g [33]. Theoretical particle size can be calculated from adsorption specific surface area data by using Equation (25).

(25) $$D\;=\;\frac{6}{\rho .S}$$

In this formula, ρ is the density of sample and S refers to the specific surface area of sample obtained from the BET method and similar to the method cited by Monshi et al. in [34]. The BET specific surface area of hydroxyapatite particles obtained from cow, pig, and chicken were 34.36 ± 0.01, 36.95 ± 0.01, and 43.39 ± 0.01 m^2^/g. As explained, a theoretical particle size can be calculated from these data and the values of crystal size for hydroxyapatite calcined at 950 °C obtained from cow, pig, and chicken were 56, 52, and 49 nm, respectively.

### 3.7. Study of TEM Analysis

**Figure 10:**
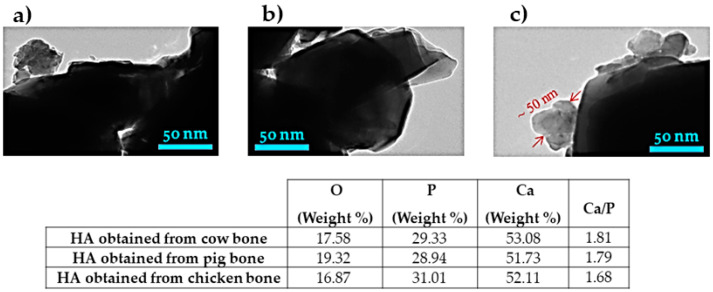
TEM images and stoichiometric composition of hydroxyapatite nanocrystals obtained from. (**a**) cow, (**b**) pig, and (**c**) chicken bones.

Figure 10 shows TEM images and stoichiometric composition of hydroxyapatite nanocrystal powders of cow, pig, and chicken bones after the ball milling process. Based on the EDX signatures, the values of ratio Ca/P for hydroxyapatite obtained from cow, pig, and chicken bones were found to be 1.81, 1.79, and 1.68, respectively. A particle may be made of several different crystallites. In addition, the TEM images show agglomerated nanosize of crystals and it is very clear that the TEM images exhibited the particle size and between all the particles, there are crystals. TEM size often matches grain size and in this case, it is apparent that some of the powder particles have nanosize and the size values are less than 100 nm (width and diameters). One single particle of about 50 nm can also be observed in chicken bone in Figure 10c clearly. Furthermore, the images seem to have irregular spherical morphology and such morphologies were cited and confirmed in reference [35]. In addition, it can be related to the deviation of data points from line equations taking account to the fit (R^2^), where there are differences between values of R^2^ in each method. According to the calculation, R^2^ allows it to be negative for some methods such as UDM (cow). It is intended to approximate the actual percentage variance, therefore, if the actual R^2^ is close to zero, the R^2^ can be slightly negative. However, the nanopowder was not dispersed perfectly, and the reason may be related to the agglomeration of powders created through the Van der Waals attraction [36]. In Figure 10, there is spacing (d_hkl_) with interplanar of less than 50 nm and the existence of hexagonal hydroxyapatite can be confirmed, according to the d_hkl_ reported in Table 1, Table 2 and Table 3. The results obtained from the methods and models are summarized in Table 9 and Table 10. In addition, the crystal size of hydroxyapatite obtained from different natural sources in several studies are presented in Table 11.

## 4. Conclusions

In this study, natural nano-hydroxyapatite was successfully prepared from cow, pig, and chicken bones. Comparison of different methods and models for the calculation of nanocrystallite size of these three bones was performed together with BET and TEM studies. In this study, data resulting from the Scherrer method while considering all peaks (straight line model) were inaccurate and above the nanoscale of 100 nm. A new model for the Scherrer method based on the straight line passing the origin is presented in this study, which results in much more accurate values when considering all or selected peaks simultaneously. An average model for each individual peak, according to the basic Scherrer formula, gives reasonable values but there is no least squares approach to decrease the sources of errors. In the Monshi–Scherrer method, if Lnβ is plotted versus Ln(1/cosθ) and the least squares method is employed, the intercept gives Ln(Kλ/L), from which a single value of L can be obtained. Plotting and calculating is easy and there is a check point for the researcher that the slope of this line must be near one, otherwise some of the erroneous data, from peaks with higher angles of diffraction, may be eliminated to increase the accuracy. The data of crystal size resulting from three models of the Williamson–Hall method are almost accurate and close together, although the setup was not like the BET experimental results, which show a finer crystal size for chicken rather than pig and cow. In addition, the values of strain and stress are compressive (positive values) in all (hkl) crystallographic lattice planes. The difference of strain values between UDM–USDM and UDEDM can be clearly related to the uniformity of deformation. The size of the crystals was obtained as 4 nm in the Halder–Wagner method, for all samples, which are much lower estimates. The lower accuracy of the H-W method can be related to the morphology of crystals, because $\frac{\beta_{\mathrm{hkl}}^{*}}{d_{\mathrm{hkl}}^{*}{}^{2}}$ linearly increases with ${\left(\frac{\beta_{\mathrm{hkl}}^{*}}{d_{\mathrm{hkl}}^{*}}\right)}^{2}$ for all reflections with a positive slope and negative intercept, showing that there are no macrostrains and also morphology of crystals are not spherical crystallite shape, confirmed through the TEM images (irregular spherical). The report of strain by the H-W method was not possible because the intercept was negative and equal to ${\left(\frac{\varepsilon }{2}\right)}^{2}$. The results gained by the SSP method were better than the H-W method, because H-W considers the contribution of low and mid angle XRD data, along with the attribution from the lattice dislocations. The strain value of cow in SSP was not reported because the intercept value was negative and is not possible for ($\frac{\varepsilon^{2}}{4})$. The final conclusion is that the Monshi–Scherrer method, for ease of use, a check point for the researcher, application of least squares method for higher accuracy, and setup of data with experimental BET results, is advised for research and industrial applications.

## Figures and Tables

**Table 1:** Crystallographic parameters of the XRD pattern related to the hydroxyapatite obtained from cow.

Cow											
2θ (Degree)	β = FWHM (Degree)	θ (Degree)	cosθ (Degree)	1/cosθ (Degree)	Ln(1/cosθ) (Degree)	β = FWHM (Radian)	Ln β (Radian)	4 sinθ (Degree)	β(Radian).cosθ (Degree)	hkl	$d_{\mathrm{hkl}}\;(Å)$
26.15	0.14	13.07	0.9740	1.02669	0.02634	0.00244	−6.0174	0.9045	0.00238	002	3.46500
28.32	0.2	14.16	0.9696	1.03135	0.03087	0.00348	−5.66072	0.9785	0.00337	102	3.17485
29.18	0.1	14.59	0.9677	1.03338	0.03283	0.00174	−6.35387	1.007	0.00168	210	3.07687
31.96	0.15	15.98	0.9613	1.04026	0.03947	0.00261	−5.94841	1.1012	0.00251	211	2.81215
32.54	0.14	16.27	0.9599	1.04178	0.04093	0.00244	−6.0174	1.1206	0.00234	112	2.78900
32.98	0.15	16.49	0.9588	1.04297	0.04207	0.00261	−5.94841	1.1353	0.0025	300	2.71354
33.97	0.14	16.98	0.9564	1.04559	0.04458	0.00244	−6.0174	1.1681	0.00233	202	2.63845
40.03	0.15	20.01	0.9396	1.06428	0.0623	0.00261	−5.94841	1.3687	0.00245	310	2.26285
46.94	0.16	23.47	0.9172	1.09027	0.08643	0.00278	−5.88387	1.5930	0.00255	222	1.94339
48.35	0.2	24.17	0.9123	1.09613	0.09179	0.00348	−5.66072	1.6377	0.00317	320	1.87176
49.73	0.15	24.86	0.9073	1.10217	0.09728	0.00261	−5.94841	1.6816	0.00237	213	1.84732

**Table 2:** Crystallographic parameters of the XRD pattern related to the hydroxyapatite obtained from pig.

Pig											
2θ (Degree)	β = FWHM (Degree)	θ (Degree)	cosθ (Degree)	1/cosθ (Degree)	Ln(1/cosθ) (Degree)	β = FWHM (Radian)	Ln β (Radian)	4 sinθ (Degree)	β(Radian).cosθ (Degree)	hkl	$d_{\mathrm{hkl}}\;(Å)$
26.12	0.13	13.06	0.9741	1.02659	0.02624	0.00226	−6.09151	0.9038	0.0022	002	3.46500
29.20	0.14	14.60	0.9677	1.03338	0.03283	0.00244	−6.0174	1.0082	0.00236	210	3.07687
32.04	0.14	16.02	0.9611	1.04047	0.03968	0.00244	−6.0174	1.1038	0.00235	211	2.81215
32.44	0.13	16.22	0.9601	1.04156	0.04072	0.00226	−6.09151	1.1173	0.00217	112	2.78900
33.07	0.14	16.53	0.9586	1.04319	0.04228	0.00244	−6.0174	1.1380	0.00234	300	2.71354
34.02	0.14	17.01	0.9562	1.04581	0.04479	0.00244	−6.0174	1.1701	0.00233	202	2.63845
40.07	0.18	20.03	0.9395	1.0644	0.06241	0.00313	−5.76608	1.3700	0.00294	310	2.26285
46.96	0.15	23.48	0.9171	1.09039	0.08654	0.00261	−5.94841	1.5937	0.00239	222	1.94339
48.34	0.14	24.17	0.9123	1.09613	0.09179	0.00244	−6.0174	1.6377	0.00223	320	1.87176
49.73	0.15	24.86	0.9073	1.10217	0.09728	0.00261	−5.94841	1.6816	0.00237	213	1.84732

**Table 3:** Crystallographic parameters of the XRD pattern related to the hydroxyapatite obtained from chicken.

Chicken											
2θ (Degree)	β = FWHM (Degree)	θ (Degree)	cosθ (Degree)	1/cosθ (Degree)	Ln(1/cosθ) (Degree)	β = FWHM (Radian)	Ln β (Radian)	4 sinθ (Degree)	β(Radian).cosθ (Degree)	hkl	$d_{\mathrm{hkl}}\;\;(Å)$
26.20	0.14	13.10	0.9739	1.0268	0.02645	0.00244	−6.0174	0.9066	0.00238	002	3.46500
28.39	0.16	14.19	0.9694	1.03157	0.03108	0.00278	−5.88387	0.9805	0.00269	102	3.17485
29.19	0.15	14.59	0.9677	1.03338	0.03283	0.00261	−5.94841	1.0076	0.00253	210	3.07687
32.03	0.16	16.01	0.9612	1.04037	0.03957	0.00278	−5.88387	1.1032	0.00267	211	2.81215
32.45	0.15	16.22	0.9601	1.04156	0.04072	0.00261	−5.94841	1.1173	0.00251	112	2.78900
33.16	0.15	16.58	0.9584	1.04341	0.04249	0.00261	−5.94841	1.1414	0.0025	300	2.71354
34.21	0.16	17.10	0.9557	1.04635	0.04531	0.00278	−5.88387	1.1761	0.00266	202	2.63845
40.05	0.17	20.02	0.9395	1.0644	0.06241	0.00296	−5.82324	1.3693	0.00278	310	2.26285
46.95	0.18	23.47	0.9172	1.09027	0.08643	0.00313	−5.76608	1.5930	0.00287	222	1.94339
48.34	0.17	24.17	0.9123	1.09613	0.09179	0.00296	−5.82324	1.6377	0.0027	320	1.87176
49.74	0.18	24.87	0.9072	1.10229	0.09739	0.00313	−5.76608	1.6822	0.00284	213	1.84732

**Table 4:** Crystallographic parameters related to the hydroxyapatite structure resulting via X’Pert software.

Bone	Crystal System	a (Å)	c (Å)	c/a (Å)	Cell Volume (Å^3^)	Crystal Density (g/cm^3^)
**Cow**	Hexagonal	9.4000	6.9300	0.7340	530.30	3.14
**Pig**	Hexagonal	9.4210	6.8930	0.7316	529.83	3.14
**Chicken**	Hexagonal	9.4210	6.8800	0.7302	528.83	3.18

**Table 5:** The (x,y) points extracted by the plots in Figure 3.

Cow		Pig		Chicken	
x	y	x	y	x	y
409.83	0.974	442.47	0.9741	409.83	0.9739
287.35	0.9696	409.83	0.9677	359.71	0.9694
574.71	0.9677	409.83	0.9611	383.14	0.9677
383.14	0.9613	442.47	0.9601	359.71	0.9612
409.83	0.9599	409.83	0.9586	383.14	0.9601
383.14	0.9588	409.83	0.9562	383.14	0.9584
409.83	0.9564	319.48	0.9395	359.71	0.9557
383.14	0.9396	383.14	0.9171	337.83	0.9395
359.71	0.9172	409.83	0.9123	319.48	0.9172
287.35	0.9123	383.14	0.9073	337.83	0.9123
383.14	0.9073	-	-	319.48	0.9072

**Table 6:** Values of crystal size extracted by the average method based on the Scherrer equation.

$\frac{K\lambda }{\beta \;cos\theta }\;\mathrm{of}\;\mathrm{Cow}$	$\frac{K\lambda }{\beta \;cos\theta }\;\mathrm{of}\;\mathrm{Pig}$	$\frac{K\lambda }{\beta \;cos\theta }\;\mathrm{of}\;\mathrm{Chicken}$
57.60	62.32	57.60
40.68	58.09	50.96
81.60	58.34	54.19
54.62	63.18	51.35
58.59	58.59	54.62
54.84	58.84	54.84
58.84	46.63	51.54
55.96	57.36	49.31
53.76	61.48	47.77
43.25	57.85	50.77
57.85	-	48.27
56	58	52

**Table 7:** Elastic compliances and stiffness constants of hydroxyapatite [26].

Elastic Compliances (GPa)					Stiffness Constants (GPa)				
C_11_	C_12_	C_13_	C_33_	C_44_	S_11_	S_12_	S_13_	S_33_	S_44_
137	42.5	54.9	172	39.6	0.88	−0.18	−0.22	0.72	2.52

**Table 8:** Young’s modulus (E) of each individual XRD pattern related to the hydroxyapatite obtained from cow, pig, and chicken bones.

Cow		Pig		Chicken	
2θ (Degree)	E (GPa)	2θ (Degree)	E (GPa)	2θ (Degree)	E (GPa)
26.15	138.889	26.12	138.889	26.20	138.889
28.32	123.935	29.20	113.636	28.39	124.121
29.18	113.636	32.04	108.694	29.19	113.636
31.96	108.734	32.44	113.02	32.03	108.684
32.54	112.887	33.07	113.636	32.45	113.054
32.98	113.636	34.02	110.706	33.16	113.636
33.97	110.598	40.07	113.636	34.21	110.733
40.03	113.636	46.96	107.155	40.05	113.636
46.94	107.161	48.34	113.636	46.95	107.154
48.35	113.636	49.73	112.702	48.34	113.636
49.73	112.571	-	-	49.74	112.734

**Table 9:** Nanosize of hydroxyapatite crystallites obtained from cow, pig, and chicken bones extracted by some calculation methods and experimental methods (BET, TEM) in this study.

Size of Crystallites	Scherrer (All Peaks/New Model/Average Model)	Monshi–Scherrer	Williamson–Hall (UDM/USDM/UDEDM)	H-W	SSP	BET	TEM
**L_cow_ (nm)**	1371/60/56	60	65/60/62	4	43	56	~ 50
**L_pig_ (nm)**	457/60/58	60	62/62/62	4	62	52	~ 50
**L_chicken_ (nm)**	196/53/52	57	65/62/65	4	57	49	~ 50

**Table 10:** Geometrical parameters of hydroxyapatite using different models in this study.

	Williamson–Hall					SSP
	UDM	USDM		UDEDM		
	Strain (ε) × 10^−4^	Stress (σ) (MPa)	Strain (ε) × 10^−4^	Strain (ε) × 10^−3^	LED (KJ/m^3^)	Strain (ε) × 10^−4^
**cow**	3	22	1.89	0.87	43.67	-
**pig**	2	18	1.59	0.73	29.70	2.83
**chicken**	4	44	3.81	0.70	28.28	4.48

**Table 11:** Reports of some studies on the nanocrystallite size of hydroxyapatite prepared at high temperature.

Number	Source	Method of Preparation	Temperature of Heat Treatment	Crystallite Phases	Size of Crystal (L) (nm)	Shape	Reference
**1**	Bovine bone	Thermal treatment	800 °C	hydroxyapatite	<100 (58 and 62)	Needle	[35]
**2**	Bovine bone	Thermal treatment	800 °C	hydroxyapatite	70–180	Irregular	[37]
**3**	Fish scale	Thermal treatment	800 °C	hydroxyapatite	30	Irregular	[38]
**4**	Bovine bone	Thermal treatment	900 °C	hydroxyapatite	30	-	[39]
**5**	Bovine bone	Thermal treatment	900 °C	hydroxyapatite	70–80	Spherical	[40]
**6**	Pig bone	Thermal treatment	1000 °C	hydroxyapatite	38–52	Rod like	[41]
**7**	Fish scale	Thermal treatment	1000 °C	hydroxyapatite	76	Nearly spherical	[42]
**8**	Clam shell	Thermal treatment	1000 °C	hydroxyapatite	53–67	Agglomerate	[43]
